# Registration and management of children with overweight by general practitioners in The Netherlands

**DOI:** 10.1080/13814788.2024.2425186

**Published:** 2024-11-14

**Authors:** Hevy Hassan, Jacoline van den Driest, Angeline Bosman, Bart Willem Koes, Patrick Jan Eugène Bindels, Marienke van Middelkoop

**Affiliations:** Department of General Practice, Erasmus MC University Medical Center, Rotterdam, The Netherlands

**Keywords:** Childhood obesity, general practitioners, primary care, overweight, weight management

## Abstract

**Background:**

General practitioners (GPs) form the gateway to healthcare in numerous European countries. Their role in addressing and managing overweight/obesity in children is crucial. In Dutch guidelines, GPs are encouraged to proactively address weight-related issues during patient consultations, regardless of the initial reason of the visit.

**Objective(s):**

To examine the frequency, management and follow-up of GP visits of children for overweight/obesity and the identification by GPs of these children presenting with other complaints.

**Methods:**

A retrospective cohort study. Health records from 2012–2021 in the Rijnmond Primary Care Database (RPCD) of children aged 2–18 with overweight/obesity who visited the GP were analysed. Children were categorised into two groups: those visiting for weight-related issues (group 1) and those visiting for other complaints but identified as overweight or obese by GPs (group 2). Data on patient demographics, reasons for contact, and management strategies were extracted.

**Results:**

From the 120,991 children, 3035 children with documented overweight or obesity were identified, 208 were excluded. The study population comprised 2827 individuals: 55% belonging to group 1, 45% to group 2. The frequency of first visits remained stable at approximately 0.5% visits per total person-years each year. Group 1 received more referrals (74%) and follow-up consultations (45.5%) than group 2 with 17% referrals and 19.7% follow-up consultations.

**Conclusion:**

This study highlights a concerning difference in the management of the two groups. Strategies for effective management of overweight in children and the GP’s role, warrant further investigation. Especially when overweight is not the primary reason for visit.

## KEY MESSAGES

Children rarely visit general practitioners for weight-related issues.

GPs recognise children with overweight but document and refer them less frequently than children who visit the GP for a weight-related issue.

Addressing overweight/obesity at the point of visit is an opportunity to improve weight control in children.

## Introduction

In many European countries, general practitioners (GPs) are the initial healthcare providers to address patients’ health problems, including overweight [1]. National and international guidelines on childhood obesity are available and consistent with growth monitoring, healthy weight, and overweight management [2,3]. Dutch guidelines for GPs recommend addressing overweight, regardless of the reason for the consultation. GPs should also provide advice on healthy lifestyles to reduce obesity and improve children’s health [4].

Typically, children under 18 visit a GP on average twice a year [5], and those with overweight tend to have more yearly visits than those with normal weight [6]. Only few children visit the GP for a weight related problem [7]. If a weight problem is not the main reason of consultation, discussions between child, parent and the GP on weight management may be hindered due to factors as time constraints, visual assessments to estimate the child’s weight, or discomfort discussing weight due to social stigma [8–10]

Earlier research shows that only 0.6% of children’s GP visits were weight-related [11]. However, it remains unclear how many children initially visit for this reason or have their weight noted during visits for other issues. This study will use electronic health records (EHRs) to outline how GPs register, manage, and follow up children with overweight in daily practice.

It is hypothesised that children rarely visit their GP with concerns about their weight, and GPs rarely address overweight during consultations when the child consults for reasons other than their weight.

Therefore, the objective of this study was twofold. Firstly, we aimed to gain more insight into the frequency at which GPs record children aged 2–18 years with overweight and obesity, as well as to explore the differences between children who initially visited their GP for weight-related issues and those who visited for other complaints but were identified as overweight or obese by their GP. Secondly, we aimed to provide insight into the management and follow-up strategies of GPs in consultations with children with overweight or obesity.

## Methods

### Study design

This retrospective cohort study used the Rijnmond Primary Care Database (RPCD), which is a derivative of the integrated Primary Care Information (IPCI) [12,13] focused on the greater Rotterdam area in the Netherlands. The RPCD includes data from approximately 120 GPs and contains pseudonymised information from the medical records of up to 500,000 primary care patients [14]. In the Netherlands, the GP is the first point of care for patients. GP files consist of medical notes, diagnostic codes, laboratory results, prescriptions, referrals and letters from secondary care, dietitians, physiotherapist and youth doctors (YD). This study was exempt from patient consent requirements due to the use of anonymised data and a waiver from the RPCD Governance Board.

### Study cohort

The study population consisted of children with overweight/obesity aged between 2 and 18 who visited their GP between 2012 and 2021. Two groups were differentiated based on the reason of consultation: those who initially (i.e. mainly/primarily) visited for weight-related issues (further referred to as group 1) and those who visited for other complaints but were also identified as overweight or obese by their GP (further referred to as group 2). To identify children with overweight/obesity in the medical file records, ICPC codes T83(Overweight)/T82(Obesity) or specific keywords were searched in the free text, including ‘Obesity’, ‘Overweight’, ‘Adipose’, ‘Worrying about weight’, and ‘Too heavy’. Children were excluded if they were identified as underweight or if negations were used in combination with keywords related to overweight or obesity. All children who were exclusively identified using ICPC T82/83 were assigned to group 1, whereas all children who were exclusively identified through free text were assigned to group 2. The algorithm was tested with samples of 100 cases by researcher (HH) and GP-researcher (JvdD) for inclusion or exclusion. The positive predictive value (PPV) for the algorithm was 99% (group 1B), and the PPV for the free text group was 95% (group 2B).

ICPC codes T82/T83 combined with other ICPC codes were manually checked by HH to confirm whether the visit was an initial visit for a weight problem (group 1A i.e. a clearly stated overweight reason for the visit or a request for help from the patient) or if the weight problem was observed by the GP while another complaint was the initial reason for the visit (group 2A i.e. if weight had been measured or observed by the GP or if it was clear that the GP had raised the issue of weight). Medical records with brief or inadequate documentation were placed in the ambiguous group. Any unclear cases were again reviewed by JvdD for the final decision on inclusion or exclusion. Cases that failed to meet the inclusion criteria for either group 1 or group 2 were subsequently excluded from further analysis.

### Data extraction

From the medical files of the study population, information on sex, age, and postal code were extracted. Children were categorised into those living in deprived areas or non-deprived areas. The Dutch Healthcare Authority (NZA) [15] defines these areas using a postal code list.

The frequency of first visits was determined by identifying newly registered children with overweight status in the EHR.

The initial reason for contact in group 1 was noted as either self-referral (i.e. visit on their own initiative) or a referral by a healthcare professional. For the children in group 2, based on the ICPC code linked to the contact, the reason for their visit was examined and categorised into eight groups based on the ICPC coding: Musculoskeletal, Respiratory, Digestive, Skin, Ear, Psychosocial, General, Other.

Additionally, from both groups 10% of the children were randomly extracted. For this 10%, the management options were manually reviewed (HH and JvdD). The GP’s management of overweight and any potential follow-up visits were recorded. The management options included ‘referral’ and ‘additional examination’. For all children, follow-up was defined as a second GP visit for weight problems within one year after the initial contact. Registration of BMI/weight was extracted.

### Statistics

Descriptive statistics were used to describe patient characteristics and frequency of first visits of the children documented as overweight or obese in the EHR. The frequency of first visits is calculated by number of first consultations of children with overweight and obesity per year and was divided by the total number of person-years (PY). PY was defined as the cumulative years in one year time of children and adolescents aged 2–18 in RPCD. The frequency of first visits were further analysed according to age group, categorised in children (2–11 years) and adolescents (12–18 years), sex and deprived area vs. non-deprived area.

A strobe checklist was employed and is presented as an additional file (Supplementary Appendix 1).

## Results

**Figure 1.**
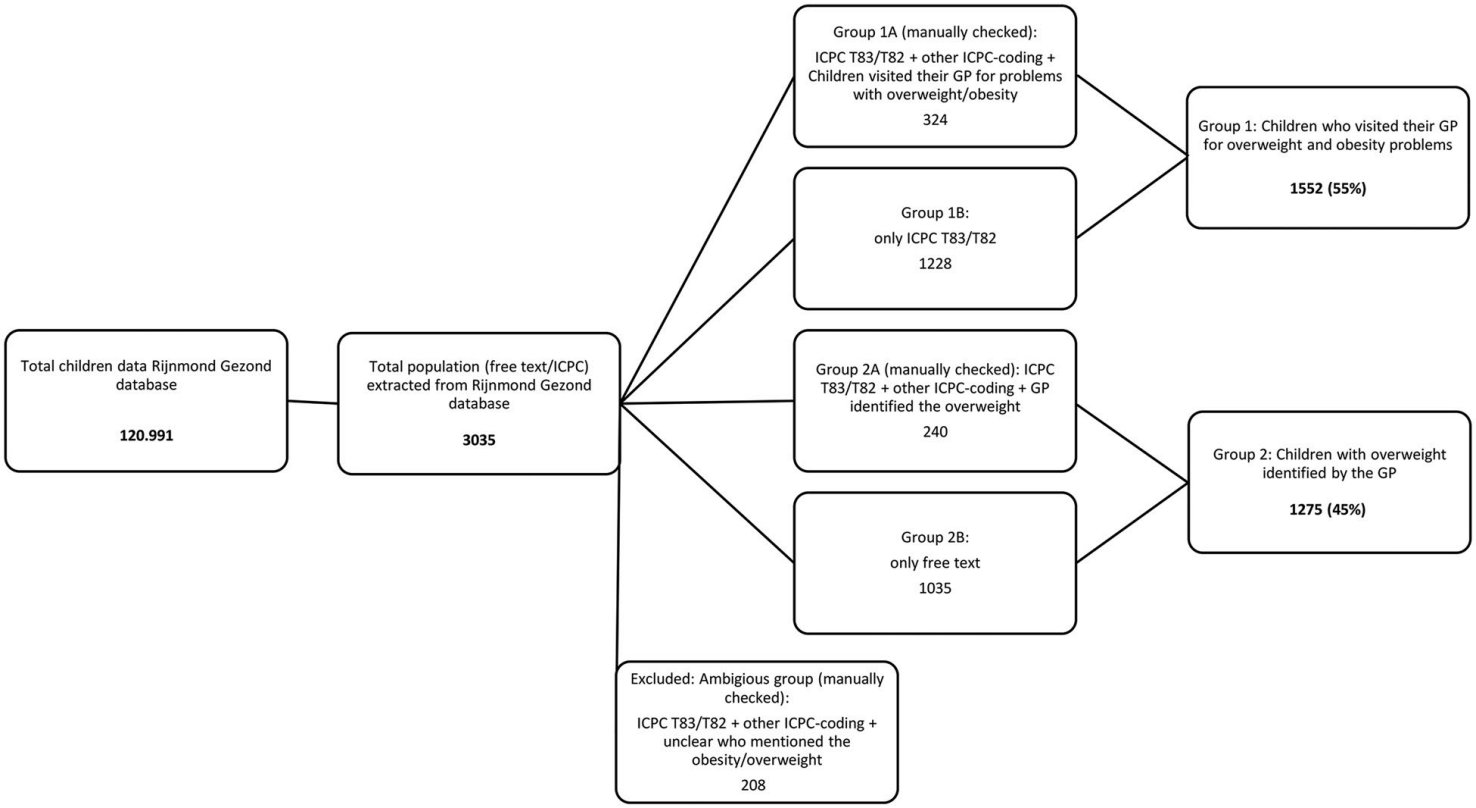
Patient selection for frequency rate of overweight or obesity of ICPC-coded T82/T83 and based on keywords in the free text.

Figure 1 shows that 3,035 children in the database (*n* = 120,991) are overweight or obese. We excluded 208 individuals who were assigned to an ambiguous category as they could not be allocated to either group 1 or group 2. The final study population comprised 2827 children and adolescents. Of these, 1552 (55%) were children initially visiting the GP for weight-related issues (group 1), 1275 (45%) visited for other complaints but were identified as overweight or obese by their GP (group 2).

Table 1 shows an overview of the population characteristics. Of the total of 2827 children, 1225 (43%) were boys, 938 (33%) lived in deprived areas, 1619 (57%) were aged 2–11 years and 1208 (43%) were aged 12–18 years. No clear differences between group 1 and 2 were observed regarding their general characteristics.

**Table 1.** Population characteristics (*N* = 2827).

	Total	Group 1 visit for weight problems	Group 2 GP identified overweight
	*N* = 2827	*n* = 1552	*n* = 1275
	*n* (%)	*n* (%)	*n* (%)
Age			
2–11 Years	1619 (57%)	944 (61%)	675 (53%)
12–18 Years	1208 (43%)	608 (39%)	600 (47%)
Sex			
Boy	1225 (43%)	640 (41%)	585 (46%)
Girl	1602 (57%)	912 (59%)	690 (54%)
Neighbourhood status			
Deprived	938 (33%)	539 (35%)	399 (31%)
Non deprived	1630 (58%)	875 (56%)	755 (59%)
Unknown	259 (9%)	138 (9%)	121 (9%)

### Frequency of first visits

For visit frequency, see Supplementary File 1. Supplementary File 1a presents first visits per 1,000 person-years and shows convergence between groups 1 and 2 over time, with a stable annual rate of approximately 0.5% newly registered overweight children. Supplementary File 1c shows that girls visit the GP more frequently for weight related issues and are more likely to be observed by the GP if they present with other complaints, compared to boys. Additionally, children in deprived areas visit their GP more often for weight-related issues and have higher rates of ­registered overweight/obesity than those in non-deprived-areas (Supplementary File 1d). In group 1, a decrease of GP visits in time for weight-related problems was seen in children aged 2–11 years (Supplementary File 1b).

### Reason for visiting the GP

**Figure 2.**
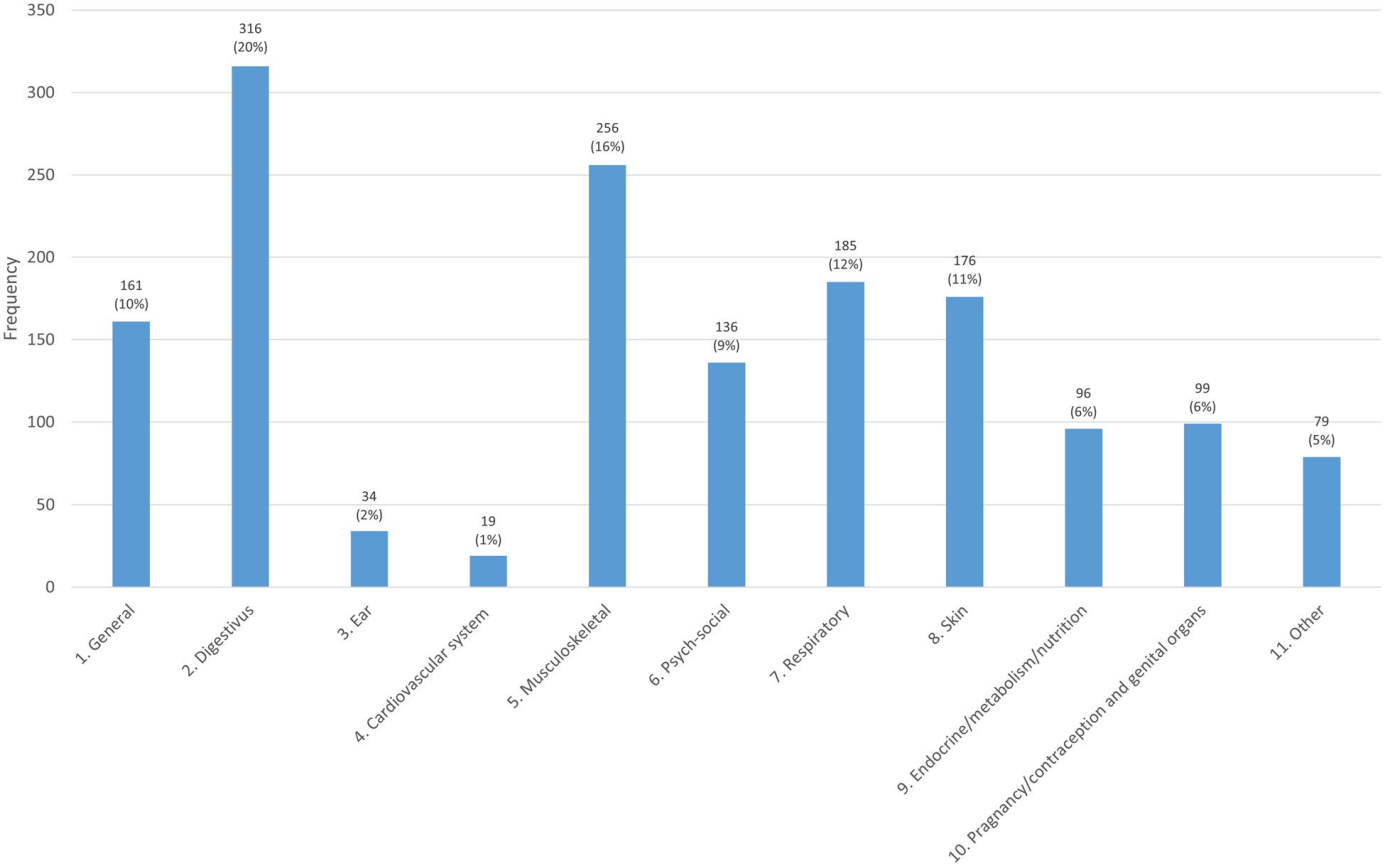
Reasons for GP consultation in which overweight was identified and registered by GP.

In group 1, 46% of the children were referred to the GP by another healthcare professional. For group 2, various reasons for the consultations were observed, with complaints of the gastrointestinal tract (20%) and musculoskeletal complaints (16%) being most frequent (Figure 2).

### Management and follow-up

Weight or BMI of the child was registered in 59% of children in group 1, while it was documented in only 28% of children in group 2. In group 1, 74% of the children were referred at first visit, compared to only 17% in group 2. Moreover, more blood tests were performed in group 1 (13%) compared to group 2 (6%) (Table 2). Most referrals were to a dietician: 58% in group 1 and 9% in group 2.

**Table 2.** Management registration by the GP during first visit.

	Total *n* = 280*	Group 1 Visit for weight problems *n* = 150*	Group 2 GP identified overweight *n* = 130*
	*N* (%)	*N* (%)	*N* (%)
Referral, total	137 (48%)	116 (74%)	21 (17%)
Dietitian	101 (36%)	90 (58%)	11 (9%)
Pediatrician	15 (5%)	12 (8%)	3 (2%)
Physiotherapist	8 (3%)	6 (4%)	2 (2%)
Psychologist	3 (1%)	1 (1%)	2 (2%)
Multidisciplinary interventions	4 (1%)	3 (2%)	1 (1%)
Other	9 (3%)	6 (4%)	3 (2%)
Additional examination^a^	28 (10%)	20 (13%)	8 (6%)

*Ten percent of each group was manually checked to determine if management documentation was recorded by the GP during the first consultation.

^a^Blood tests (TSH, glucose).

A higher percentage of children in group 1 (45.5%) received follow-up consultation with their GP compared to group 2 (19.7%). No differences in follow-up consultations were observed in age, sex, or neighbourhood.

## Discussion

### Main findings

The frequency of first visits for children with overweight or obesity was comparable between group 1, where the initial reason for the visit was weight-related issues, and group 2, where overweight was identified by the GP. However, in children in group 1 more referrals and follow-up consultations were registered compared to children in group 2.

### Comparison with existing literature

About 0.5% new visits per total person-years were annually recorded as overweight or obese by GPs for children aged 2–18 years, meaning 5 out of every 1,000 children were registered each year. The Netherlands Institute for Health Services Research (NIVEL) reported [16], an incidence of 4.4 per 1000 PY for overweight (T83) and 8.1 per 1000 for obesity (T82) among adults and children [16]. As no separate data were presented for children it is not possible to compare our findings with these data. Moreover, our study was performed in the Rijnmond region, known to have a relatively higher obesity rate [17]. A previous Dutch study showed that only 0.6% of children consulted a GP for overweight or obesity concerns [11]. The authors suggest that this relatively low number may be attributed to the fact that children may not proactively seek advice from their general practitioner regarding obesity [7]. This aligns with our findings, which are marked by a similarly low frequency of new consultations in our study’s group 1. While group 1 has informative data, there is insufficient focus on the documentation for children in group 2. The WHO European Childhood Obesity Surveillance Initiative (COSI) reports that one in three children in the WHO European region is overweight or obese [18]. Additionally, studies have shown that overweight children tend to visit their general practitioner (GP) relatively more often than their peers without overweight [6]. However, in line with our study, research showed that GPs do not regularly record weight in their registry system during consultation [19], and reporting rates may be relatively low due to various factors such as health system disparities, diagnostic criteria, and registry systems [19,20]. Dutch obesity guidelines for GPs emphasise the crucial role of GPs in identifying overweight or obese individuals, regardless of the purpose of the visit, to initiate secondary prevention [21]. The majority of children in group 2 did not receive the ICPC classification for overweight/obesity, suggesting that although GPs recognise the issue, they do not use the ICPC classification.

Children in group 1 received a substantial higher number of referrals (74% vs. 17%) and follow-up appointments (45.5% vs. 19.7%) than whose initial visit was not weight-related. One of the reasons for this difference may relate to the severity of the overweight, that may differ between these groups. Though, as only weight data were available in 59% of the cases in group 1 and 28% of the cases in group 2, it is not possible to make definitive conclusions on this matter as we have not analysed this data due to its limitations. Secondly, since the measurement of height, weight, and BMI is not a standard practice for GPs in the Netherlands, it is plausible that they may even have overlooked overweight children due to the challenges associated with visually estimating a child’s weight (group 2) [8,9]. Thirdly, it is uncertain whether discussions about overweight actually took place in group 2. The barriers identified by literature based on qualitative studies may impede GPs from initiating discussions about overweight resulting in the low numbers of ICPC codes in group 2 [4,10,22]. GPs may face barriers related to time constraints, stigma and fear of discussing eating disorders, or even scepticism about the relevance of this topic or the utility of their role in addressing it [8–10,23,24]. Finally, the low referral and follow-up rates at consultation may be due to the common practice of providing lifestyle advice first, or the motivation level of the child or parents [25]. However, our data does not include information on these details.

It was noticeable that when GPs did refer children, they tend to refer them to a dietician, even though literature and (European) guidelines advice a multidisciplinary approach [4,26]. This preference may be due to the limited availability of easily accessible and effective multidisciplinary programmes for both children and parents in their neighbourhood. Future research should concentrate on the role of the GP, the expectations of GPs and the practicality of implementing new approaches in everyday practice.

The frequency of visits for different patient characteristics exhibit subtle variations. In the Netherlands, while there is no difference in the prevalence of obesity between boys and girls in the open population, girls are registered more often, possibly because they are more dissatisfied with their weight than boys [27,28]. Children in a deprived area have a higher frequency of visits than those in non-deprived areas, possibly due to higher overweight prevalence in these regions [29,30]. Additionally, schools in the greater Rotterdam area promote healthy lifestyles, potentially leading to increased self-initiated actions by parents and children [31,32]. In group 1, a negative trend was seen in the frequency of first visits over time in children aged 2–11 years. This may be due to the introduction of the new Youth law in the Netherlands, which authorised specialised youth doctors (YD) to directly refer children to specialists [33]. However, the referral numbers by YD are unknown. The trend may also reflect a decrease in overweight among children aged 4–11 years in the overall population between 1990 and 2021 [28].

### Strengths and limitations

To the authors’ knowledge this is the first study to assess how often GPs record children aged 2–18 years with overweight and obesity and to distinguish between children who initially visited their GP for weight-related issues versus children who visited for other complaints but were identified as overweight or obese by their GP.

The most significant limitation of this study is that the primary purpose of EHR is not data collection, which means the reliability of the data relies on the GP’s use of ICPC-codes and note-taking. We attempted to identify children with overweight using keywords in free text, but it is possible that we missed some patients. Nevertheless, we believe that our cohort was as comprehensive as possible.

Another limitation was that the existing database did not contain standardised information regarding the weight of all registered children, making it impossible to distinguish between overweight and non-overweight individuals. In only 45% of the children weight, length, or BMI were recorded in the EHR. Consequently, the absence of weight and height data for all children precluded the ability to ascertain the prevalence of overweight and obesity among children.

This study was constrained by the absence of a clearly defined threshold for deprived area. Furthermore, individual socioeconomic status data were unavailable within the consultation dataset.

For management and follow-up purposes, data were randomly selected, with 10% of the children from each group being chosen for examination. This method was chosen due to the labour-intensive nature of the manual review process, which was carried out by two project members, making it impractical to review all 2827 cases. This methodology aligns with those used in previously published studies. Considering the randomness of the selection, it is improbable that the results would have been markedly different had the entire study population been reviewed.

## Conclusion

This study reveals that children who visit the GP for weight-related issues receive more referrals and follow-up than those who visit for other reasons, highlighting the need to address children with overweight/obesity in general practice. Further research should clarify the role of the GP in addressing children with overweight and obesity.

## Supplementary Material

Supplemental Material

Supplemental Material
