# Supplementary figures and images for: Registration and management of children with overweight by general practitioners in The Netherlands
Source: Eur J Gen Pract. 2024 Nov 14;30(1):2425186. doi: 10.1080/13814788.2024.2425186 (PMC11565679; doi:10.1080/13814788.2024.2425186)

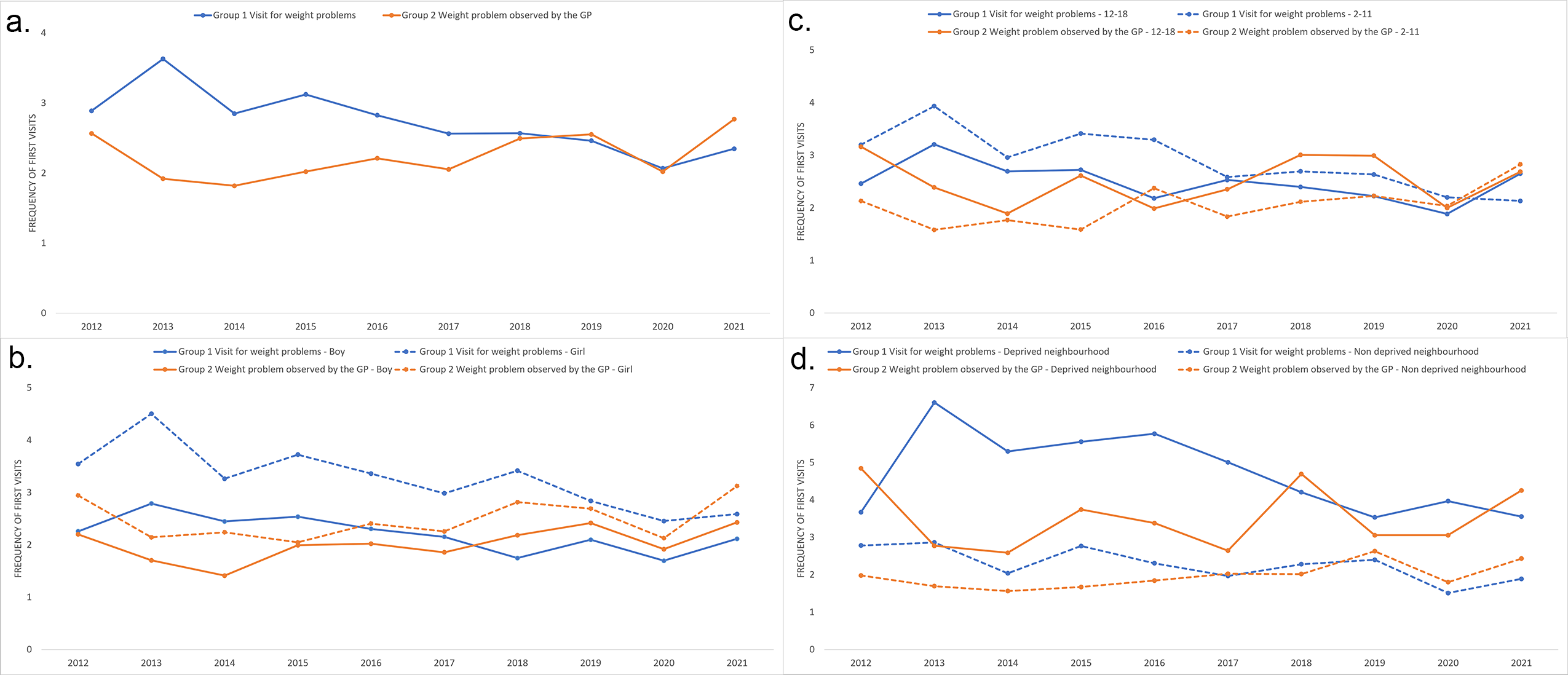

Supplement: Supplemental Material [file IGEN_A_2425186_SM0469.tif]
